# Large-scale investigation for antimicrobial activity reveals newly-identified defensive species across the healthy skin microbiome

**DOI:** 10.1038/s41467-026-73524-z

**Published:** 2026-05-25

**Authors:** Uyen Thy Nguyen, Rauf Salamzade, Shelby Sandstrom, Mary Hannah Swaney, Elizabeth C. Townsend, Sherrie Y. Wu, J. Z. Alex Cheong, Joseph A. Sardina, Isabelle Ludwikoski, Mackinnley Rybolt, Hanxiao Wan, Caitlin M. Carlson, Jordana Ferro, Owen McArthur, Won Se Suh, Robert Zarnowski, David R. Andes, Cameron R. Currie, Lindsay R. Kalan

**Affiliations:** 1 Department of Medical Microbiology and Immunology, School of Medicine and Public Health, University of Wisconsin-Madison, Madison, Wisconsin USA; 2 Microbiology Doctoral Training Program, University of Wisconsin-Madison, Madison, Wisconsin USA; 3 Department of Biochemistry and Biomedical Sciences, McMaster University, Hamilton, Ontario, Canada; 4 M. G. DeGroote Institute for Infectious Disease Research, McMaster University, Hamilton, Ontario, Canada; 5 David Braley Centre for Antibiotic Discovery, McMaster University, Hamilton, Ontario, Canada; 6 Department of Bacteriology, College of Agriculture and Life Science, University of Wisconsin-Madison, Madison, Wisconsin USA; 7 Department of Medicine, Division of Infectious Disease, School of Medicine and Public Health, University of Wisconsin-Madison, Madison, Wisconsin USA

**Keywords:** Microbiome, Antifungal agents, Antimicrobials

## Abstract

The skin microbiome forms a protective barrier to pathogens, including through the production of antimicrobial metabolites. Here, we present EPIC^HHS^, a large and taxonomically diverse skin microbiome culture collection of 968 strains from eight body sites. EPIC^HHS^ captures >95% of cumulative species-level abundance across 268 skin metagenomes. It includes isolates present at <0.1% relative abundance and the cultured representatives for eight species not previously isolated, markedly expanding current skin microbiome resources. A contact-independent screen assaying ~14,000 pairwise interactions against 22 pathogens revealed widespread antagonism with striking enrichment for antifungal activity. Finally, functional genomic analysis, including 287 EPIC^HHS^ isolate genomes, demonstrated a diverse landscape of skin-associated biosynthetic gene clusters that are mostly uncharacterized. Together EPIC^HHS^, its functional and genomic characterization, establishes the skin microbiome as a reservoir for specialized metabolism and provides a platform for microbiome-based antimicrobial discovery.

## Abstract (web summary)

Authors present the EPithelial Isolate Collection (EPIC), a large skin microbiome culture collection, uncovering new species, widespread antifungal activity, and hidden biosynthetic diversity, revealing insights into the skin’s potential for microbiome-driven antimicrobial discovery.

## Introduction

Human skin represents a first line of defense against mechanical and chemical insults while maintaining homeostasis^1–7^. One of the most significant roles of the skin is to act as a barrier to invading pathogens. This physical barrier is fortified by a diverse microbiome composed of bacteria, fungi, viruses, and microeukaryotes^8^. The surface area of the skin is estimated to be around 30 m^2^, including appendages such as hair follicles and sweat ducts^5^, making it one of the most expansive direct host-microbe interfaces of the body. Across the body, unique microenvironments form on the skin with characteristic moisture levels, pH, and lipid content, driving the composition of the associated microbial community^7,9–12^. Far from mere bystanders, members of the skin microbiome help maintain the integrity of the skin barrier through both direct and indirect defense mechanisms^9–11^.

Constituents of the skin microbiome can serve roles as defensive symbionts, defined as microorganisms that form mutually beneficial relationships with their host by providing protection against pathogenic colonization, such as through the production of antimicrobial molecules. Efforts to mine the human microbiome for genes encoding bioactive metabolites have revealed a rich biosynthetic potential^13^. Given that bacteria inhabit different niches within the human body and are exposed to different competitors in the environment, we expect that skin microbiota produce metabolites that are relevant to the niche that they colonize. An example is lugdunin, a non-ribosomal thiazolidine cyclic peptide targeting methicillin-resistant *S. aureus* produced by *Staphylococcus lugdunensis*.

The identification and characterization of antimicrobial molecules produced by skin microbiota has historically focused on easily cultivated organisms, particularly intraspecies competition within the *Staphylococcal* genus^14–20^. Studies of common and abundant skin microbes show they harbor a wide diversity of biosynthetic gene clusters (BGCs) in their genomes^21–23^; however, this likely represents just the tip of the iceberg. The skin microbiome also contains numerous species of lower abundance^24^, including some discovered in just the last five years, exemplified by the recent Skin Microbial Genome Collection (SMGC)^24^. Given that currently characterized BGCs come from a limited set of microbial genera and most BGCs remain uncharacterized^25,26^, the skin microbiome represents a potentially rich, untapped source of new therapeutic molecules, including those with antimicrobial properties.

**Fig. 1:**
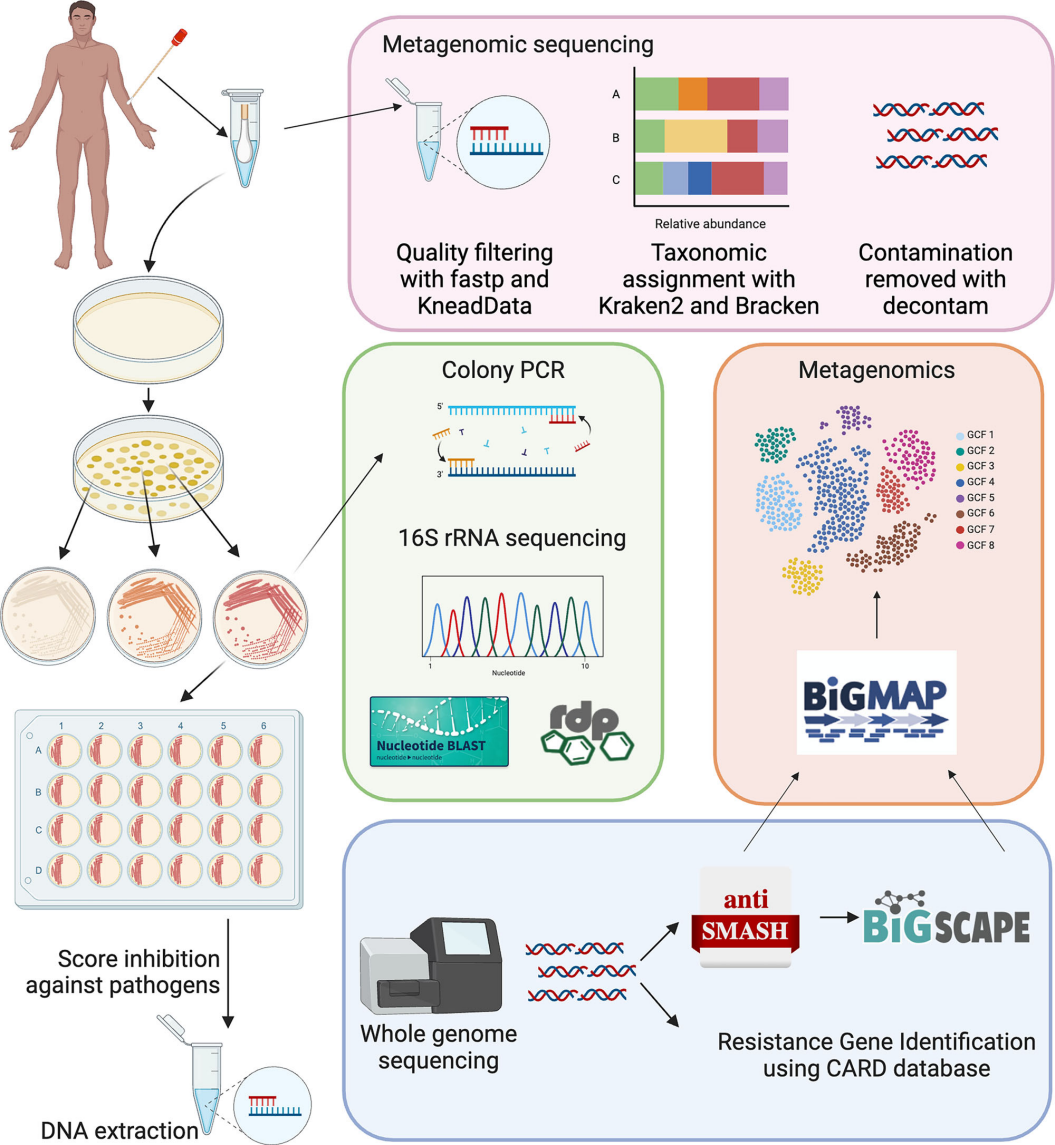
Comprehensive workflow for skin microbiome sampling and genomic analysis. Skin samples were collected from each participant from eight different body sites for metagenomic sequencing. A second set of samples was collected for strain isolation. Metagenomic sequencing was performed with taxonomic assignments using Kraken2 and Bracken. For strain isolation, samples were plated on selective media to isolate pure bacterial colonies and identified by 16S rRNA gene sequencing or whole genome sequencing. Skin isolates were assessed for antimicrobial activity using a large-scale solid-phase biological assay. Biosynthetic gene clusters and gene cluster families were annotated with antiSMASH/BiG-SCAPE and BiG-MAP for genomes and metagenomes, respectively. Antibiotic resistance genes were called using the Resistance Gene Identifier (RGI) and the Comprehensive Antibiotic Resistance Gene (CARD) database. Created in BioRender. Kalan, L. (2026) https://BioRender.com/i3esbew.

We have developed the EPithelial Isolate Collection (EPIC), a microbial biorepository of >6000 bacterial isolates from 1060 mammalian microbiome swabs. This collection is derived from epithelial sites, including the skin^22,27–29^, oral^30,31^ and nasal^27,32^ barriers in humans, swine^33^, non-human primates, horses, goats, donkeys, and cows. Here, we detail the extensive characterization of the subset of healthy human skin isolates included in EPIC, that we call EPIC^HHS,^ along with matched metagenomes from eight skin sites. EPIC^HHS^ encompasses 968 bacterial isolates, 287 with genomes, and 268 metagenomes (Fig. 1). Our collection is one of the largest and most diverse collections^24^, comprising hundreds of rare and low-abundance strains^24,34^. We assessed antagonistic traits between isolates and a panel of 22 diverse pathogens, including Gram-negative, Gram-positive and fungal species. Finally, we further charted the diverse biosynthetic landscape encoded within isolate genomes and described four newly identified species, unique to this collection, three of which exert significant antifungal activity. This rich and diverse isolate collection enables systematic exploration of skin microbiome chemistry, illuminates the breadth of antagonistic interactions within the skin microbiome, and will provide new molecular insights into how commensal bacteria defend against expansion of potential pathogens.

## Results

**Fig. 2:**
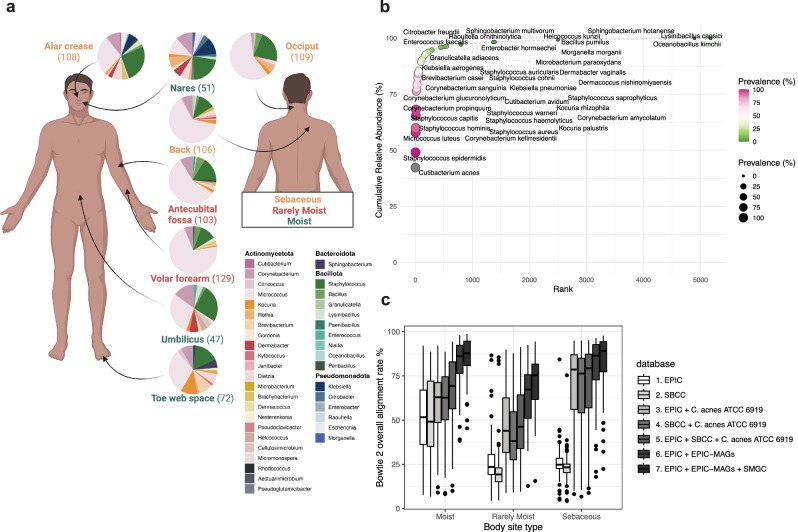
Strain library identification at each body site. **a** Individual pie charts represent the community structure at each site. Pie labels indicate body site, font colors indicate body site type (sebaceous, moist, rarely moist), and the n-value of each pie chart is shown in parentheses. Bacterial composition is shown within the pie charts. Created in BioRender. Kalan, L. (2026) https://BioRender.com/w8g8aa0. **b** Ranked species (x-axis) based on their cumulative sum of mean metagenomic relative abundance (y-axis) of species detected in the skin metagenomes and culture collection. Each dot represents a specific species, with its color and size reflecting the percent prevalence across the metagenomic dataset. The gray dot indicates *C. acnes* cumulative mean abundance, a dominant skin species only detected in the metagenome. A threshold of 0.01% for abundance was applied to determine species presence. **c** Alignment rate of metagenomic readsets (y-axis) to various skin-associated genome collections (color-coded) categorized across body site types (x-axis). The two largest, right-most, genome collections include MAGs. There were 268 metagenomic readsets in total; 68 were from rarely-moist body sites, 99 were from moist body sites, and 101 were from sebaceous body sites. Center lines show the median, box bounds represent the 25^th^ and 75^th^ percentiles, and whiskers demonstrate 1.5 × interquartile range (IQR).

EPIC^HHS^ is derived from 34 healthy volunteers sampled from 8 distinct body sites encompassing moist (nares, umbilicus, toe web space), rarely moist or dry (antecubital fossa and volar forearm), and sebaceous (alar crease, back, and occiput) microenvironments (Fig. 2a, Supplementary Dataset 1). Skin swabs from 17 participants were cultured, while shotgun metagenomic sequencing was performed for all 34 participants (268 samples) to assess microbiome agreement between metagenomes and the culture collection. This resulted in 968 skin-associated bacterial strains from 136 human skin samples. The composition of EPIC^HHS^ offers a resource that expands and complements prior isolate collections^24^. For example, 55.9% of the isolates, and 74.3% with genomes are classified outside of the skin-dominant genera *Staphylococcus* and *Corynebacterium*, compared to 23.1% of isolates in the Skin Bacterial Culture Collection (SBCC)^24^. Furthermore, EPIC^HHS^ contains skin isolates belonging to an additional 24 genera not represented in the SBCC (Supplementary Dataset 2).

### The EPIC^HHS^ library includes rare, low-abundance, and taxonomically diverse bacteria

To maximize the phylogenetic diversity of EPIC, skin swabs were cultured using multiple media types, including with components to suppress *Staphylococcus* growth and to foster the growth of *Corynebacterium* and lower abundance Actinomycetota, which are prolific producers of natural products^35^. In total, the 968 skin isolates represent at least 90 species spanning 40 genera^36^. Most isolates fall within the phylum Actinomycota (*n* = 523), followed by Bacillota (*n* = 171), Pseudomonadota (*n* = 27), and Bacteroidota (*n* = 4). *Micrococcus, Staphylococcus*, and *Corynebacterium* species were most frequently isolated. We also isolated rare and phylogenetically diverse species across different genera, such as *Kocuria, Aestuariimicrobium, Kyotococcus, Nesterenkonia, Microbacterium, Brachybacterium, Rothia, Dietzia*, and *Dermabacter* (Supplementary Datasets 2, 3). Distinct compositions of bacterial isolates were derived from each site, with sampling of the nares contributing the greatest taxonomic diversity, where 51 isolates belonging to 17 different genera were collected (Fig. 2a).

Metagenomic sequencing confirmed concordance between culture-dependent versus culture-independent profiling (Fig. 2b, c). Similar to previous findings^37–39^, *Cutibacterium, Corynebacterium, Micrococcus*, and *Staphylococcus* are the most abundant skin colonizers across all sampled body sites. Overall, the culture collection represents >95% of the cumulative mean species abundance across all 268 skin metagenomes (Fig. 2b). Focusing on low-abundance genera, defined as a relative abundance of <1% in metagenomes, EPIC^HHS^ isolates show strong representation of these taxa. The proportion of isolates per genus is significantly positively correlated with metagenome proportions (Spearman rho > 0.5, *p* < 0.05) across most body sites, including the back, occiput, nares, toe web space, and antecubital fossa (Supplementary Fig. 1).

Because purely aerobic culturing conditions were used, EPIC^HHS^ is deficient in anaerobic *Cutibacterium* species, therefore we mapped metagenomic readsets to EPIC^HHS^ isolate genomes with the addition of the *C. acnes* ATCC 6919 genome. High mapping rates, especially at moist and sebaceous sites, indicate that the culture collection captures a substantial fraction of dominant skin-associated taxa in these environments (Fig. 2c). Including genomes for isolates from the SBCC further increased mapping rates. To further characterize genomic diversity and account for unmapped reads, we assembled the metagenomic reads and recovered 1,326 sample-specific metagenomic assembled genomes (MAGs), which were dereplicated to 226 species-distinct MAGs (Supplementary Dataset 4). Of these, 25 represent candidate species-level lineages not matching species in either the SMGC genome catalog or EPIC^HHS^ isolate genome collection and were classified as being at least medium-quality. Adding the EPIC^HHS^-derived MAGs, together with SMGC MAGs, further improved mapping rates, particularly in rarely moist sites. Overall, in ~74% of metagenomic samples, more than 75% of reads mapped to the combined reference database of EPIC^HHS^isolate genomes, EPIC^HHS^MAG and SMGC genomes.

### A pairwise interaction screen reveals widespread inhibition of pathogens by skin-associated bacterial strains

**Fig. 3:**
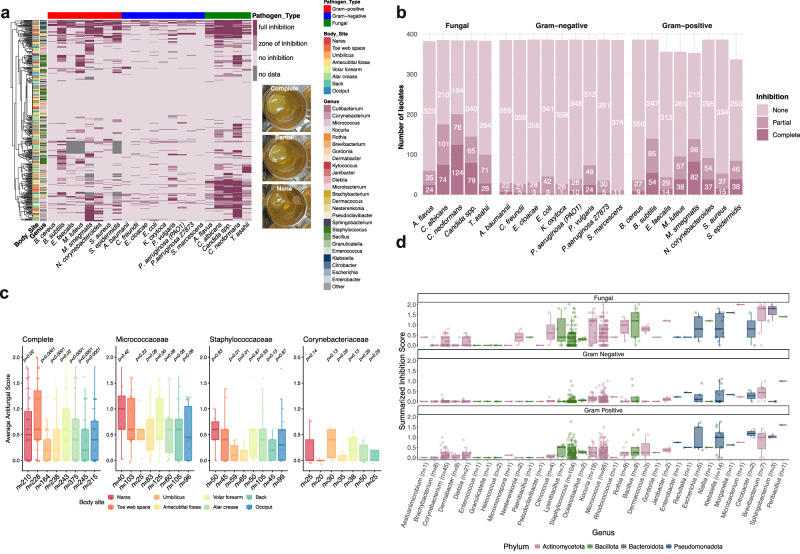
Bacteria from the human skin exhibit specific and broad antifungal activity. **a** Hierarchical clustering of pairwise interactions where skin bacteria are tested against a panel of pathogens. Growth patterns of pathogens are scored from 0 (no inhibition) to 2 (complete or full inhibition). Rows represent individual isolates annotated by body site and genus. Columns represent a single pathogen grouped by Gram-positive, Gram-negative, and fungal species. Examples for each inhibition profile: complete (top), partial (middle), and none (bottom). **b** Quantification of inhibition (none [light pink], partial, and complete [dark pink]) profiles. The y-axis shows the number of isolates, and the x-axis lists members of the pathogen panel. **c** Summarized antifungal inhibition scores (y-axis) for each body site (x-axis) for the complete dataset (first panel), along with subset scores for isolates from the *Micrococcaceae* (second panel), *Staphylococcaceae* (third panel), and *Corynebacteriaceae* (fourth panel) families. Statistical comparisons were performed against the toe web space as the reference group using a two-sided Wilcoxon rank-sum test with exact *p*-values. The Benjamini-Hochberg method was used to adjust for multiple comparisons. Adjusted *p*-values are labeled. **d** Inhibition scores of genera in EPIC^HHS^ against fungal (top), Gram-negative (middle), and Gram-positive (bottom) pathogens. Color indicates phylum of isolates. The sample size of cultivated isolates regarded for each genus is shown in parentheses. Center lines show the median, box bounds represent the 25^th^ and 75^th^ percentiles, and whiskers demonstrate 1.5 × IQR.

We developed a pairwise interaction screen to assess contact-independent inhibition of human pathogens (Fig. 3a; Supplementary Dataset 5). From the collection, 615 randomly selected isolates were co-cultured on solid media with a panel of 22 Gram-positive and Gram-negative bacterial and fungal pathogens (13,530 total interactions). Interactions are scored as no, partial, or complete inhibition (Fig. 3a). To ensure data quality, we removed profiles with insufficient or contaminated growth of the ‘producer’ or in any of the 22 ‘pathogen’ growth control wells. We then filtered redundant isolates, defined as the same species from the same host sample or genomes with >99% ANI and identical inhibitory profiles, yielding 386 unique inhibitory profiles. Hierarchal clustering revealed widespread inhibition of fungi, and to a lesser degree Gram-positive pathogens, while antagonism towards Gram-negative pathogens was less common (Fig. 3a).

Given the prominence of fungal inhibition, we quantified the spectrum of isolates inhibiting at least one of the five fungal pathogens. *Cryptococcus neoformans* was the most susceptible, with 124 isolates fully inhibiting growth, compared to 74 and 79 isolates inhibiting *Candida albicans* and *Candida* sp., and 24 and 28 isolates had full inhibition against *Aspergillus flavus* and *Trichosporon asahii*, respectively (Fig. 3b). Over 30 isolates broadly inhibited *C. neoformans*, *Candida* sp., and *C. albicans*, while six inhibited all five fungal pathogens tested (Supplementary Fig. 2). To assess activity against multidrug-resistant *Candida auris*^40–43^, we tested a random subset of 51 isolates with strong *C. albicans* activity. Of this subset, 35% of the isolates, spanning diverse genera, displayed partial to complete inhibition of *C. auris* (Supplementary Dataset 6). Antifungal inhibition also varied by body site, where isolates from the toe web, a niche enriched for fungal diversity, display elevated antifungal activity compared to other body sites. At a granular level, antifungal inhibition scores of the major taxa in the *Micrococcaceae*, *Staphylococcaceae*, and *Corynebacteriaceae* families demonstrate that the *Micrococcaceae* are driving this pattern, including species in the genera *Micrococcus*, *Kocuria*, and *Rothia* (Fig. 3c). We next calculated a summarized inhibition score across pathogen groups. Genera exhibiting higher antifungal activity compared to antibacterial activity included *Staphylococcus* and *Micrococcus* (Fig. 3d), as well as genera that are common to skin but often low-abundance such as *Kocuria*, *Microbacterium*, *Brevibacterium*, and *Sphingobacterium*.

### The EPIC^HHS^ expands the known biosynthetic potential of the skin microbiome

**Fig. 4:**
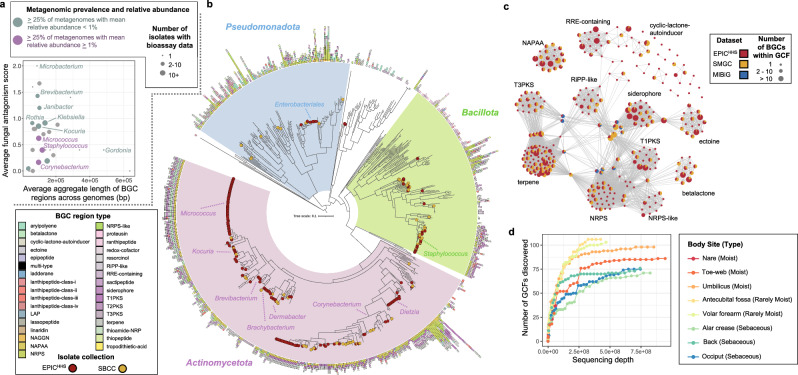
Charting the biosynthetic potential of the skin microbiome. **a** Average fungal antagonism scores (y-axis) and per-genome biosynthetic content for genera in EPIC^HHS^. Size indicates the number of isolates with bioassay data. Color illustrates whether the individual genus is found in greater than 25% of metagenomes with a mean relative abundance of greater or lesser than 1%, respectively. **b** A comprehensive phylogeny of SMGC and EPIC genomes, with genomes of cultured isolates marked (EPIC^HHS^ = red, SBCC = gold). Stacked bar plots depict BGC type counts. **c** A network of 305 GCF (nodes) identified in the genomes using BiG-SCAPE. Edges indicate common antiSMASH-based BGC types shared between GCFs. Node size corresponds to the number of BGC instances belonging to a GCF and the source dataset. RiPP = ribosomally synthesized and post-translationally modified peptide, RRE = RiPP recognition element, NRPS = non-ribosomal peptide synthetase, T1PKS = type 1 polyketide synthase, T3PKS = type 3 polyketide synthase, NAPAA = non-alpha poly-amino acid. **d** Cumulative GCF discovery by BiG-MAP is plotted as a function of aggregate sequencing depth at each body site across multiple individuals using skin metagenomes from Swaney et al. 2022. Metagenomes are ordered from lowest to highest sequencing depth.

To explore the vast biosynthetic potential suggested by widespread antimicrobial activity, we sequenced the genomes of 287 isolates representing diverse species in the EPIC^HHS^ collection and dereplicated genomes at 99% average nucleotide identity^44^, resulting in 182 genomes for BGC annotation^45^. After predicting BGCs, we found that many genera exhibiting high antifungal activity are low-abundance actinomycetes that are prevalent across skin metagenomes. Carriage of biosynthetic traits in these genera appears selective, with individual genomes often carrying fewer than 200 kb of BGCs per genome (Fig. 4a). To more comprehensively explore the diversity of BGCs in skin-associated microbes, BGCs were predicted from 621 isolate genomes or bacterial MAGs from the SMGC, a recently established reference collection of genomes from the skin microbiome^24^. Mapping the 3183 predicted BGCs onto a species phylogeny of the source genomes revealed that many skin-associated microbes possess a broad range of different BGC types, including non-ribosomal peptide synthetases (NRPSs), polyketide synthases (PKSs) and terpenes (Fig. 4b). To further assess biosynthetic diversity, comprehensive clustering of BGCs across EPIC^HHS^ and SMGC genomes was performed, resulting in 1960 distinct gene-cluster families (GCFs)^46^ (Fig. 4c, Supplementary Datasets 7, 8).

Of the 305 GCFs identified within EPIC^HHS^ skin isolate genomes, only 12 GCFs (3.9%) correspond to experimentally characterized BGCs from the MIBiG database, which links BGCs to known metabolites (Supplementary Dataset 9). This includes BGCs for the synthesis of aureusimines^47^ and dehydroxynocardamine^48^ from *Staphylococcus* and *Corynebacterium*, respectively. While some of the 305 GCFs found in EPIC^HHS^ genomes are also present in the SMGC, the majority, 54.4%, spanning at least 30 types of BGCs from 28 genera, are unique to EPIC^HHS^ genomes (Fig. 4b). We further assessed whether BGCs from EPIC^HHS^ and SMGC genomes match GCFs in the BiG-FAM database, which catalogs and has clustered over a million BGCs (Fig. 4c). Of the 305 GCFs found in EPIC^HHS^ genomes, 31 (10.2%) featured a highly distinct domain composition (*T* > 1500) to established GCFs in BiG-FAM (Supplementary Fig. 3a).

While BGC type classification at the genomic level cannot infer function, given the results from bioactivity co-culture screening, we expected a fraction of predicted BGCs to underlie synthesis of metabolites with antibacterial or antifungal activity. To infer the potential uniqueness of such BGCs, we investigated the resistome of the skin metagenome, focusing on encoding genes that may confer self-protection to known antibiotics. Resistance to macrolides and beta-lactams was the most common; however, overall, a low prevalence of antimicrobial resistance (AMR) genes within the skin metagenome was observed (Supplementary Fig. 4a). Similarly, through assessing whole genomes of cultured isolates, only 41 (22.5%) of the 182 genomes encoded antibiotic resistance protein homologs. However, when considering resistance conferred by specific protein residues, such as intrinsic alleles of ribosomal protein RpsL contributing resistance to aminoglycosides, 122 of 182 genomes contained at least one resistance determinant (Supplementary Fig. 3b, Supplementary Dataset 10).

To understand if particular body sites exhibit greater biosynthetic potential, we examined the distribution of predicted GCFs in our skin metagenomes (Supplementary Fig. 4b, c)^49^. We also assessed the number of distinct GCFs discovered as a function of sequencing depth at each body site using rarefaction^50,51^ (Fig. 4d, Supplementary Fig. 4d). Sebaceous sites showed the clearest saturation for GCF discovery, consistent with their high abundance of *C. acnes*, a species known to encode a limited set of GCFs^8,15,22,38^. In contrast, dry sites generally exhibited the steepest rate of GCFs discovered as a function of sequencing depth and did not reach saturation, consistent with their higher taxonomic variation^27,52,53^, potentially from transient or environmentally derived microbes. To test this possibility, we annotated host-associated protein domains and found that isolates from dry sites had lower proportions of proteins with these domains (*p* = 3.68e-4; Wilcoxon rank sum; Supplementary Fig. 3c), indicating a greater presence of environmental microbes at these sites^54^. However, BGCs of isolates from dry sites were also enriched for proteins with such domains compared to the rest of their genomic contexts (*p* = 4.57e-8; Fisher’s exact test), similar to a comprehensive test involving isolates from every body site (*p* = 5.26e-28; Fisher’s exact test; Supplementary Fig. 3d). These observations are in accordance with the perspective that traits, such as specialized metabolites, can be associated with specific ecological settings, even if the species encoding for their synthesis are broadly distributed across multiple environments^55–57^.

### Discovery of previously uncharacterized skin bacterial species with strong antifungal activity

**Fig. 5:**
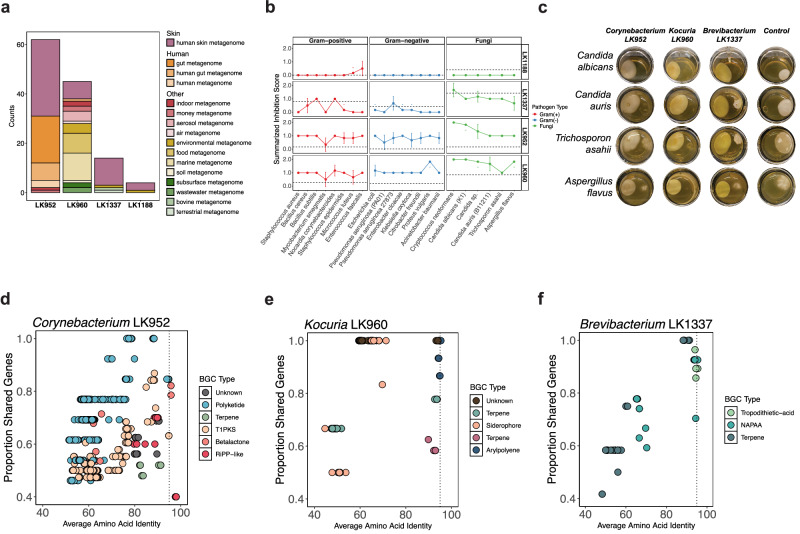
Identification of newly-identified skin species. **a** Branchwater analysis of NCBI SRA metagenomes with query genomes of the newly-identified species shows associations with human skin metagenomes. Colors indicate the metagenome type each genome was found in. **b** Biological activity of *Aestuariimicrobium* LK1188 (first), *Brevibacterium* LK1337 (second), *Corynebacterium* LK952 (third), and *Kocuria* LK960 (fourth) using co-culture screening. Solid line indicates inhibition scores against Gram-positive (red), Gram-negative (blue) and fungal pathogens (green). Dotted line indicates the average inhibition score of all isolates tested in each corresponding genus against each pathogen type. Mean ± SD, *n* = 6 (3 biological × 2 technical replicates). **c** Pairwise interaction between (in columns) LK952 (first), LK960 (second), LK13337 (third), and pathogen control (fourth) against fungal pathogens (in rows) *C. albicans* (first), *C. auris* (second), *T. asahii* (third), and *A. flavus* (fourth). Investigating BGC novelty of **d** *Corynebacterium* LK952, **e** *Kocuria* LK960, and **f** *Brevibacterium* LK1337 to representative genomes from each genus. 1108 *Corynebacterium*, 93 *Kocuria*, and 97 *Brevibacterium* genomes were used to reference the skin species. Colors indicate the BGC type predicted in each species. The x-axis indicates the average amino acid identity of a single BGC identified compared to the target genome. The y-axis indicates the proportion of genes in identified BGC co-located in the target genome.

EPIC^HHS^ isolates with genomes were taxonomically classified against the Genome Taxonomy Database (GTDB) and the SMGC, identifying three *Corynebacterium* isolates and one *Brachybacterium* isolate predicted as novel species in the SMGC. Until now, these species lacked cultured representatives (Supplementary Fig. 5, Supplementary Dataset 3). In addition, four newly-identified species isolates not represented in either the GTDB or the SMGC were discovered. Each belongs to a distinct but known genus: *Aestuariimicrobium*, *Corynebacterium*, *Kocuria*, and *Brevibacterium* (Supplementary Dataset 3). To assess their uniqueness, we searched public metagenomes in the NCBI Sequence Read Archive to determine their environmental distribution^58,59^. *Corynebacterium* isolate LK952, *Aestuariimicrobium* isolate LK1188, and *Brevibacterium* isolate LK1337 are most commonly detected in human skin metagenomes while ~fifteen percent of the metagenomes *Kocuria* isolate LK960 was identified in corresponded to human skin, supporting the skin as a bona fide niche for these species (Fig. 5a).

Consistent with our data showing genera in the *Micrococcaceae* family are antifungal, the *Kocuria* isolate completely inhibits *C. albicans*, *C. auris*, and *A. flavus* and partially inhibits *Trichosporon asahii*. The newly-identified *Corynebacterium* species exerts moderate antifungal activity, while the newly-identified *Brevibacterium* isolate displays less antifungal activity, and *Aestuariimicrobium* has no antifungal activity (Fig. 5b, c). Prediction and annotation of BGCs using both antiSMASH and GECCO revealed these newly-identified species encode one to four BGCs each (Supplementary Fig. 6).

Finally, we assessed the novelty of each species’ biosynthetic capacity with the program abon^60^ by querying the presence of their BGCs in publicly available genomes of their respective genera. This comparative analysis confirmed that BGCs encoded in each predicted species genome largely have <95% amino acid identity to homologous BGCs from related species in their respective genera (Fig. 5d–f).

## Discussion

Here, we present a human skin microbiome collection, including hundreds of strains, original cultured isolates for at least 8 species, and metagenomes from 34 participants, including 25 MAGs predicted to be novel. The isolate collection is several times larger, adds diversity to existing resources, and was designed to include taxa that are rare or low in abundance. Using this collection, we tested hundreds of antagonistic interactions against 22 pathogens. After filtering and assessing 8333 pairwise interactions, we discovered broad antimicrobial activity, most notably antifungal activity, in hundreds of strains. This indicates an extensive antimicrobial biosynthetic potential, which we then explore in individual genomes, describing an untapped resource of uncharacterized BGCs across multiple genera. Our collection thus has significant potential for the expansion of our knowledge of biosynthetic resources within the skin microbiome and provides a platform for exploring this resource.

Intraspecies antagonism on the skin has been reported for select lineages^61^. Our findings reveal broader antagonistic networks across taxonomic lineages. Previously uncharacterized skin isolates primarily spanning four bacterial phyla illustrate extensive cross-kingdom antimicrobial capacity. Broadly, we find taxa within the *Micrococcaceae* family are driving observed fungal inhibition, with isolates from moist body sites showing the greatest antifungal activity. For example, 87.5% of *Kocuria* isolates and 77.8% of *Micrococcus* isolates from the toe web space inhibit *C. albicans*. In the literature, there is evidence that *Micrococcus* species produce antifungal factors, however elucidation of chemical structures is lacking^62^. Despite limited studies, there is also evidence that isolates belonging *Kocuria* species may inhibit fungi via secreted factors and small molecules^63,64^. Together, this suggests that inter-kingdom antagonism may be as significant as intraspecies competition in shaping microbial community assemblages on human skin.

Eleven isolates are representative of eight novel species belonging to distinct but known genera, including *Aestuariimicrobium*, *Corynebacterium*, *Kocuria*, *Brevibacterium*, and *Brachybacterium*. Three of the newly-identified species in the genera *Corynebacterium*, *Kocuria*, and *Brevibacterium* inhibit human fungal pathogens, and their BGCs exhibit genetic variation relative to other species from their genera. Thus, compared to the extensively studied filamentous actinomycetes, which have been heavily mined^35,65^, the BGCs from low-abundant genera on the skin have received less attention but are potentially valuable sources of structurally distinct metabolites. To further assess genomic diversity represented in skin metagenomes, we generated MAGs. A subset corresponded to EPIC^HHS^ isolates, many matched SMGC-reported genomes, and a third subset lacked close matches to any database. The modest overlap between isolate genomes and MAGs emphasizes the sensitivity of culture to recover low-abundance taxa that lack sufficient metagenomic coverage for assembly. Nonetheless, we find EPIC^HHS^ isolates account for >95% of cumulative species-level abundance across metagenomes and incorporating MAGs into our reference database results in high sample-level metagenomic read mapping, indicating additional diversity from low-abundant or uncultured lineages.

The skin microbiome encodes broad classes of BGCs, expanding the catalog of BGCs in the human microbiome^13^. Across sequenced isolates, we identified at least 30 BGC types and 305 distinct GCFs, spanning diverse skin microenvironments, revealing a rich reservoir of specialized metabolites. Predicted terpenes and betalactone BGCs were consistently abundant, suggesting these natural product classes are integral to skin ecology. Terpenoids constitute the largest and most diverse class of natural products with highly diverse and broad biological activities. Betalactones, on the other hand, represent a smaller class of BGCs with only 30 core structures reported as of 2018^66^; however, genome mining continues to reveal new compounds like the antifungal Alligamycin A^67^.

Most GCFs identified in EPIC^HHS^ genomes were distinct from previously-characterized BGCs^24^, underscoring substantial unexplored biosynthetic diversity. The CARD Resistance Gene Identifier (RGI) was used to detect self-resistance to known antibiotics. RGI uses curated CARD reference models to detect antimicrobial resistance gene homologs, even in distantly related, non-pathogenic species, by recognizing conserved sequence features rather than requiring exact matches^68^. Searching for self-resistance genes has been used strategically for the discovery of known natural product classes and target identification. Consistent with reports that healthy host-associated microbiomes carry few AMR genes^69,70^, this highlights that skin-derived antimicrobials may act through previously uncharacterized mechanisms, and there is a need to functionally characterize these molecules and their mode of action.

This study has limitations. While this resource is one of the most diverse human barrier site isolate collections, *Micrococcus* is overrepresented. They are prevalent members of the skin microbiome, strict aerobes, and non-fastidious^71^. To reduce bias in our genomic analysis, we performed genomic dereplication while preserving strain-level variation, considering that strain-level variants can have functional differences, owing to different auxiliary genes and BGCs^72^. Despite the overrepresentation of some genera, our culture collection correlates strongly with metagenome proportions and successfully captured fastidious bacteria, including previously undescribed, rare, and low-abundance skin species that appear at less than 1% abundance in metagenomes (Supplementary Fig. 1)^24,27,37,38^.

Another limitation of this study is the challenge of definitively linking secreted metabolites with their corresponding BGCs. Although we attempted to connect bioactivity with BGC content, this was only feasible at the individual strain-level due to the substantial experimental effort required. As a result, large-scale functional pairing of BGCs and metabolites remains an important future direction, ideally through high-throughput approaches. It is also possible that BGC diversity may not correspond to true chemical and functional novelty, thus downstream experimental validation of predicted BGCs and biological activity will be critical. Many of the skin-derived Actinomycetota are understudied and require physiologically relevant conditions for growth (e.g., solid media, sweat or sebum, and temperature sensitivity), reflecting nutrient limited conditions on the skin. It is possible that skin microbes may rely less on de novo biosynthesis and more on precursor biotransformation or community interactions. A lack of genetic tools in many of these genera further impedes manipulation. Future work will benefit from heterologous expression systems and optimizing skin-relevant growth conditions to prioritize and characterize key BGCs.

Human skin is a critical defense barrier, hosting a unique microbiome that can produce specialized metabolites to protect its niche and, in turn, the host. This study revealed a large and phylogenetically diverse set of bacterial species with significant antifungal activity and expands our understanding of antagonistic interactions occurring on the skin. While the broad ecology and colonization resistance function of the skin microbiome is well documented, specific mechanisms and defense against fungal pathogens are less understood. This is a critical gap given that fungi are implicated in numerous dermatological conditions, while also existing as skin commensals^3^. Our findings open new lines of investigation for the discovery of structurally unique and safe antifungal compounds. Future studies directly examining the ability of skin-microbiome-derived strains to prevent pathogen colonization on the skin surface will be crucial to confirm the inhibitory activity observed in vitro and to define the mechanisms that could enable their translation into therapeutic strategies.

## Methods

### Participant recruitment

We recruited participants at the University of Wisconsin-Madison under an Institutional Review Board-approved protocol. Inclusion criteria included age >18 yr. No formal exclusion criteria were applied. A total of 34 participants were enrolled (22 female, 11 male, age range 20–62 years). Informed consent was obtained from all participants prior to sample collection. Participation in the study was completely voluntary, and participants were able to stop at any time. Participants received no payment for being a part of the study.

### Sample collection

We collected samples for metagenomic sequencing by wetting a sterile foam swab in nuclease-free water and swabbing approximately a 1 in. x 1 in. area of the selected site on the right-hand side of the participant’s body. We swabbed the area approximately 15 times in a downward motion with constant pressure while rotating the swab. We collected the swab into a 2.0 ml BioPure Eppendorf tube containing 300 μl Lucigen MasterPure™ Yeast Cell Lysis solution. Tubes were labeled with subject ID, body site, visit number, and date of collection and stored at −80 °C until DNA extraction.

For culturing samples, we used the Copan Diagnostics ESwab. The swab was wet in nuclease-free water, and the sample was taken from approximately a 1 in. × 1 in. area of the selected site on the left-hand side of the participant’s body. We swabbed the area approximately 15 times in a downward motion with constant pressure while rotating the swab. We then placed the swab into the Copan Diagnostics ESwab collection tube, which contained 1 ml of liquid Amies media. We labeled the tubes with subject ID, body site, and date of collection and stored them at 4°C for up to 24 h until processing.

### Strain isolation and storage

Within 24 hours of collection, we added 100 μl of the Amies media from the culture sample collection tube to 900 μl of sterile water to create a 1:10 dilution. We then transferred 100 μl of the diluted sample to each of 3 agar plates: Brain Heart Infusion (BHI) + 50 mg/L mupirocin, BHI + 0.1% Tween 80 + 50 mg/L mupirocin, and Trypticase Soy Agar with 5% Sheep’s blood (blood agar) + 50 mg/L mupirocin. Blood agar plates were purchased premade, and we spread 750 μl of 1 mg/ml mupirocin to the top of the agar and allowed them to soak into the media. We distributed the diluted sample evenly across the plate using sterile glass beads. We incubated the inoculated plates at 28 °C for 48 to 72 h, depending on colony formation. After incubation, we chose distinct colonies based on color, size, morphology, and opacity and struck onto a new BHI + 0.1% Tween 80 plate. We grew these strains for 24 h or until fully grown. If the isolate was not a pure culture, we replated the isolate until it was a pure culture. Once a pure culture was obtained, we inoculated an overnight liquid culture in 3 ml BHI + 0.1% Tween 80. We stored our strains long-term at −80 °C in a 2.0 mL cryotube by combining 900 μL overnight liquid culture with 900 μL 30% glycerol.

### Strain library

Of the strains isolated in our study, 451 underwent 27 F 16S rRNA gene Sanger sequencing (Functional Biosciences, Madison, WI; see below). We classified sequences to genus-level using either RDP or NCBI Blast. We identified 279 isolates with whole genomes to the species level by running their genomes through autoMLST.

### Colony PCR

We performed Colony PCR on the overnight liquid culture of 451 isolates in a 25 μL reaction containing 12.5 μL EconoTaq® PLUS 2X PCR Master Mix by Lucigen, 1 μL of 10 μM 27 F 16S rRNA primer, 1 μL of 10 μL 1492 R 16 s rRNA primer^73^(Integrated DNA Technologies, Coralville, Iowa, USA), 10 μL nuclease-free water, and 0.5 μL of the overnight liquid culture. We amplified the 16S rRNA gene using the following settings: initial denaturation at 95 °C for 10 min, followed by 30–40 cycles of 95 °C for 30 s, annealing at 54 °C for 30 s, and extension at 72 °C for 60 s, with a final extension at 72 °C for 5 min and a hold at 4 °C indefinitely. We confirmed amplification of the 16 s rRNA gene by gel electrophoresis.

### 16S sanger sequencing

We cleaned the PCR product using the Sigma-Aldrich GenElute PCR Clean-Up kit, following the kit directions. We submitted a clean PCR product to Functional Biosciences in Madison, WI for Sanger sequencing of the 27 F end. Quality-trimmed FASTA files were inputted into the RDP Classifier for genus-level identification.

### Microbiome DNA extractions

Microbiome DNA extractions were performed on a set of swabs collected into 300 uL Lucigen Master-Pure Yeast Cell Lysis buffer and frozen at −80 °C. Samples were thawed on ice prior to extraction. Extraction methods and data were incorporated from Swaney, Sandstrom and Kalan 2022^27^. Metagenomic profiling was also similarly performed using Kraken2. Metagenomic coverage of species diversity across different skin site categories was performed using Bowtie 2 (v2.5.4) alignment to various genome sets through assessment of logging information for the overall alignment rate.

### Bioassay

Fresh bacterial isolates were struck from isolation plates or from freezer stock. We tested the bioassays in 12-well plates containing 3 mL BHI solid agar in each well. A single colony from each skin isolate was streaked in a half-moon shape onto the left half of each well of two 12-well plates. We incubated the plate for 7 days at 28 °C. After 7 days, we spotted 3 µL of a 1:10 dilution of overnight liquid cultures from 22 different pathogens onto the right side of the well. Two 12-well control plates with media along were included as a positive control for pathogen growth. Plates were again incubated for 7 days before scoring inhibition of growth on a scale from 0 to 3.

To increase throughput, we adapted the bioassay method to using 24-well plates in the spring of 2020. Liquid overnight cultures of skin isolates are grown in 3 mL BHI + 0.1% Tween 80. The bioassays were done in 24-well plates containing 1.5 mL 0.5X BHI + 0.1% Tween solid agar in each well. We spotted 1.5 µL of the liquid overnight skin isolate culture onto the left half of the well. Plates were incubated at 28 °C for 5 to 7 days. Slow-growing isolates were inoculated on day 0, while fast-growing isolates were introduced on day 2. On day 7, we inoculated 1 µL of 1:10 diluted overnight pathogen cultures onto the right side of each well. Pathogens include Gram-positive bacteria (*Bacillus cereus*, *Bacillus subtilis*, *Enterococcus faecalis*, *Micrococcus luteus*, *Mycobacterium smegmatis*, *Staphylococcus aureus*, *Staphylococcus epidermidis*), Gram-negative bacteria (*Acinetobacter baumannii*, *Citrobacter freundii*, *Enterobacter cloacae*, *Escherichia coli*, *Klebsiella oxytoca*, *Proteus vulgaris*, *Pseudomonas aeruginosa* PAO1, *Pseudomonas aeruginosa* 27873, *Serratia marcescens* 8055), and fungi (*Aspergillus flavus*, *Candida albicans* K1, *Candida* sp., *Cryptococcus neoformans*, *Trichosporon asahii*, and *Candida auris* B11211). Plates were incubated at 28 °C for 3 days. After 3 days of incubation, skin isolates were scored as before. We applied a coarse filter for bioassay quality by first removing 70 strains with inadequate pathogen growth in control wells across the pathogen panel. Subsequently, we filtered for strain redundancy by removing 118 duplicate strains from the same host sample. Of which, 71 belong to the genus *Micrococcus* and 47 are without identification. 29 isolates with genomes were removed by genome dereplication. Lastly, 12 isolates were removed due to contaminated stocks, resulting in a discrepancy in identification. After filtering, 386 isolates remained for downstream analysis.

The scoring scale for all plate formats is as follows; 0 – No inhibition, 1 – Slight inhibition, or more transparent than control, 2 – Medium inhibition, or zone of inhibition, 3 – Full inhibition, no pathogen growth. Inhibition scores were further simplified, grouping slight and medium inhibition together. The simplified scores are as follows: 0 – No inhibition, 1 – Slight inhibition, 2 – Full inhibition. Photos were taken of each well and uploaded along with scores to a database. The summarized inhibition score was calculated by summing the inhibition scores of each isolate against each pathogen group and dividing by the number of pathogens tested

### gDNA extractions for whole genome sequencing

We extracted bacterial gDNA from plated isolates using the Sigma-Aldrich GenElute Bacterial Genomic DNA Kit. Library preparation and whole-genome sequencing on Illumina NextSeq 550 were performed at the SeqCenter sequencing facility (Pittsburgh, PA, USA).

### Genome assembly, representative selection through dereplication analysis, and phylogeny construction

We processed sequencing data for quality and adapters using fastp (v0.20.0) with parameters “--detect_adapter_for_pe -f 20”. Subsequently, short-read assemblies were constructed using Unicycler (v0.4.7) with default settings. Standard assembly statistics, including N50, were computed using the abyss-fac program in ABySS (v2.3.9) with the default options to only consider contigs greater than 500 bp in length. Assembly completion and contamination were further assessed using CheckM2 (v1.0.1).

Dereplication of the full set of 287 isolate genome assemblies was performed using a cutoff of 99% identity using dRep (v3.2.2)^44^ with parameters “--S_algorithm fastANI -sa 0.99” led to the selection of 182 distinct representative genomes (Supplementary Dataset 2). Genomes were taxonomically classified using GTDB-Tk (v1.7.0)^36^ with GTDB release 202. GTDB-Tk was unable to assign species designations for 11 genomes. To further assess whether these genomes corresponded to newly-identified species, we computed their average nucleotide identity (ANI) comparing to genomes in the recently established SMGC database^24^ using FastANI (Supplementary Dataset 3). Six of the genomes featured >95% ANI and >20% coverage to genomes from the SMGC and were thus regarded as known species. Genomic dereplication further indicated that two of the five remaining genomes represented the same species belonging to the genus of *Kocuria*. progressiveMAUVE (build date May 15 2023) was used to visualize genomic similarities between EPIC isolate genomes and MAGs of novel species identified by Kashaf-Saheb et al. 2022.

A phylogeny of 644 genomes from the EPIC^HHS^ (*n* = 182) and SMGC (*n* = 462) genomes was created based on multiple sequence alignment of universal ribosomal proteins using GToTree (v1.6.36). GToTree removed 159 SMGC genomes from inclusion in the final phylogeny for lacking too few instances of the expected universal ribosomal proteins. The phylogeny was midpoint-rooted for visualization.

A set of 526 Pfam domains previously found to be eukaryotic host-associated in three or more bacteria taxa^54^ was used to annotate coding sequences in EPIC^HHS^ genomes using PyHMMER (v0.11.0).

Sample-specific metagenomic-assembled genomes were constructed from processed metagenomic reads using an approach similar to what Saheb-Kashaf et al. 2022 used to construct the SMGC (Supplementary Dataset 4). Briefly, metaSPAdes (v4.2.0) was used for metagenomic assembly with the option “--only-assembler” requested to improve computational efficiency. Afterwards, MetaWRAP (v1.3.2) was used to automate binning through running the modules “binning” and “binning_refinement” with the options “--maxbin2 --metabat2 --concoct -t 10 -m 64” and “-c 50 -x 10” applied, respectively. Finally, MAGs were extracted, and subsequently CheckM2 was used to assess completeness and contamination, AbySS was used to compute N50 and genome-size, and GTDB-Tk was used to taxonomically classify them based on GTDB R202. Similar to Saheb-Kashaf et al. 2022, we also assessed the presence of ribosomal and transfer RNAs using INFERNAL cmsearch (v1.1.5) and tRNAScan-SE (v2.0.12), respectively, and used a similar approach to them in categorizing MAGs based on quality. For high-quality MAGs, we required <5% contamination and >90% completeness estimates by CheckM2, an N50 of ≥ 50 kb, the presence of at least 18 tRNAs, and the presence of all three rRNAs. For medium-quality MAGs, we required <10% contamination and >50% completeness estimates by CheckM2 and an N50 of ≥ 10 kb. All other assemblies were regarded as low-quality. Metagenomic assemblies and MAGs generated for this study can be found on Zenodo at: https://zenodo.org/records/18882798.

### Annotation of biosynthetic gene clusters, determination of gene cluster families and subsequent profiling in metagenomes

antiSMASH (v6.0.0) was used to predict the presence of BGCs for our dereplicated set of isolate genomes as well as metagenomic assembled genomes (MAGs) from skin microbiomes by Kashaf et al. 2022 using the parameters: “--taxon bacteria --genefinding-tool prodigal --fullhmmer --asf --cb-general --cb-subclusters --cb-knownclusters --cc-mibig --rre --pfam2go”. Following BGC annotation, we grouped analogous BGCs across genomes into GCFs using BiG-SCAPE(v1.1.5)^46^ with requests for inclusion of singletons, hybrid classifications to be turned off, inclusion of MIBiG reference BGCs, and a mixed analysis to be performed. A network graph with GCFs as nodes was constructed through parsing BiG-SCAPE mixed clustering results and linking nodes based on shared annotations for GCFs stemming from antiSMASH type classifications of member BGCs. Visualization of the network was performed using igraph with the layout “layout_nicely”. BiG-SLICE (v1.1.1)^74^ in query mode was used to assess the similarity of BGCs from EPIC and SMGC genomes to cataloged BGCs in the comprehensive BiG-FAM database. The program visualize_BGC-Ome.py in the *lsa*BGC suite was used for creating visualizations of BGC schematics for novel species. To more comprehensively assess the biosynthetic potential of newly-identified isolates, GECCO (v0.9.6) was additionally applied for BGC annotation; however, all hits overlapped with antiSMASH annotations. BiG-MAP (version committed on March 22, 2023)^49^ was used to profile the presence of BGCs from the analysis in 268 metagenomes from Swaney et al. 2022. We ran BiG-MAP with default parameters except for BiG-MAP.family.py, where a distance cutoff of 0.3 was requested for BiG-SCAPE-based grouping of GCFs (default in BiG-SCAPE) instead of 0.2 (default in BiG-MAP) to use similar criteria for delineating GCFs in BiG-MAP and BiG-SCAPE analyses. GCFs were regarded as present in a single metagenome if their full coverage was ≥ 50%, full normalized RPKM was ≥ 0.1, core gene coverage was ≥ 75%, and core gene normalized RPKM was ≥ 3.0. These parameters were selected based on manual examination of observed distributions (Supplementary Fig. 4b). Using these parameters, 158 different GCFs were identified by BiG-MAP in at least one metagenome. These corresponded to 148 distinct GCFs in the original BiG-SCAPE analysis, with ten BiG-SCAPE GCFs having two representatives regarded as separate GCFs by BiG-MAP.family.py. Discordance is likely because BiG-MAP performs a preliminary collapsing of similar BGCs using MASH and runs BiG-SCAPE with only representative BGCs from this initial clustering. For rarefaction analysis of GCF discovery as a function of metagenomic sequencing depth, we referenced coarser BiG-SCAPE GCF designations of representative gene clusters measured by BiG-MAP to avoid inflated metrics of GCF discovery. Comprehensive annotation for BGCs of raw metagenomic assemblies from metaSPAdes was also performed using antiSMASH with the options: “--taxon bacteria --genefinding-tool prodigal-m --fullhmmer”. Afterwards, BiG-SCAPE was run on the antiSMASH BGC predictions using the options “--mibig --include_singletons --mix” to determine GCFs and a rarefaction analysis was carried out to measure GCF discovery across metagenomic samples from different body sites.

To predict the collection of antibiotic resistance (AMR) genes, the antibiotic resistome of the human skin microbiome, we used the Comprehensive Antibiotic Resistance Database’s (CARD) Resistance Gene Identifier (RGI) software (v6.0.1). Briefly, we surveyed both isolate genomes and metagenomes using RGI to assess the presence of known AMR resistance genes in the CARD database. Filtered metagenomic short read sequences were compared to the reference AMR sequences in CARD via the k-mer alignment (KMA) algorithm. Metagenomes were considered to have the AMR gene present if metagenomic reads covered greater than or equal to 70% of the reference CARD AMR sequence and MAPq score greater than or equal to 50. AMR genes were grouped by the class of drugs they conferred resistance to, and if a gene conferred resistance to multiple drugs, it counted toward both. Prevalence for antimicrobial resistance across each body site was calculated (number of subject samples from the body site with a gene confirming resistance to an antibiotic divided by the total number of subject samples from the site [*n* = 34]).

To assess whole bacterial isolate genomes, we searched for “Perfect” and “Strict” matches of AMR genes in the CARD. We further required the percentage length of the reference sequence and identity of matches to be ≥90%. We grouped AMR genes by the class of drugs they conferred resistance to, and if a gene conferred resistance to multiple drugs, it counted toward both. The prevalence of antimicrobial resistance within the bacterial isolates from each species was calculated by the number of isolates, with at least one gene confirming resistance to an antibiotic, divided by the total number of species isolates assessed.

### Assessment of newly-identified species distributions across public metagenomes and comparison of their biosynthetic content to BGCs from known species in their respective genera

For each of the four species identified, the Branchwater webserver (https://branchwater.sourmash.bio/; accessed October) was used to identify public metagenomes from NCBI’s SRA database which feature them. To regard a species as present in a metagenome, we required a cANI value of at least 95% to our query genome, a threshold commonly used to delineate species, and a containment value of at least 0.5. The abon program, within the zol suite (v1.3.11), was used with default settings to assess the conservation of BGCs from each species across genomes for other species belonging to the same genus. Comprehensive databases were independently set up for each genus by gathering genomes belonging to them from GTDB R202.

### Statistical analysis

To check whether representative EPIC genomes from rarely-moist sites (*n* = 54) had lower proportions of proteins featuring host-associated domains than moist or sebaceous sites (*n* = 128), a one-sided Wilcoxon rank sum test was used. Pairwise comparison with antifungal bioassay scores between the toe web space and all other body sites was performed using two-sided Wilcoxon rank-sum tests with Benjamini-Hochberg adjustment for multiple comparisons. A one-sided Fisher’s exact test was used to assess whether BGC regions featured a greater proportion of proteins with host-associated domains in comparison to genomic contexts. Correlations using the mean relative abundance of individual genus per body site between the metagenomics dataset and culture isolate collection were assessed using Spearman’s rank correlation coefficient.

### Reporting summary

Further information on research design is available in the Nature Portfolio Reporting Summary linked to this article.

## Supplementary information

Supplementary Information

Description of Additional Supplementary Files

Supplementary Dataset 1

Supplementary Dataset 2

Supplementary Dataset 3

Supplementary Dataset 4

Supplementary Dataset 5

Supplementary Dataset 6

Supplementary Dataset 7

supplementary dataset 8

supplementary dataset 9

supplementary dataset 10

Reporting Summary

Transparent Peer Review file

## Source data

Source data

## Data Availability

The genomic assemblies generated for isolates in this study have been deposited in NCBI under BioProject PRJNA803478. The metagenomic sequencing data generated in this study have been deposited in the Sequence Read Archive (SRA) under BioProject PRJNA763232. Metagenomic assemblies and MAGs can be found on Zenodo 10.5281/zenodo.18882798. Source data are provided with this paper.

## Code availability

Code used for analyses and figures is available on GitHub (https://github.com/Kalan-Lab/SkinBioassayStudy)^75^. The libraries ggplot2 and igraph in R, the iTol webserver, and BioRender were the primary software used for visualization.
