# Supplementary material for: Large-scale investigation for antimicrobial activity reveals newly-identified defensive species across the healthy skin microbiome
Source: Nat Commun. 2026 May 25;17:6806. doi: 10.1038/s41467-026-73524-z (PMC13385363; doi:10.1038/s41467-026-73524-z)
Supplement: Supplementary file 1 — Supplementary Information [file 41467_2026_73524_MOESM1_ESM.pdf]

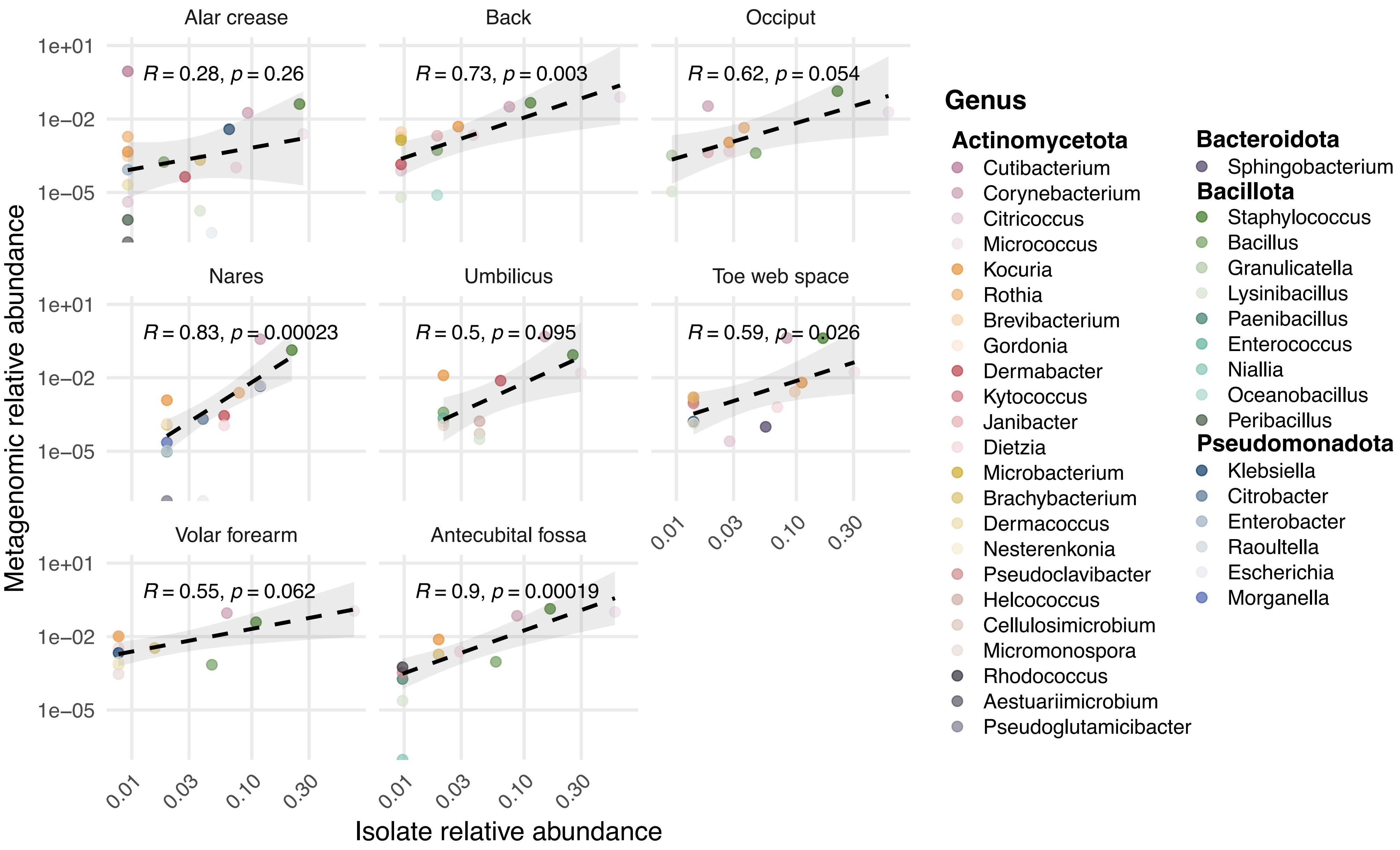

Supplementary Figure S1: Cultured genera detection in skin metagenomic data. Scatterplot for bacterial genera (color-coded) abundance shared between skin metagenomes (y-axis) and cultured isolates (x-axis) is shown. Each panel is labeled with the body site name, where panels in the top row indicate sebaceous sites, middle rows indicate moist sites, and bottom rows indicate rarely moist sites. Spearman's rank correlation coefficient was used to detect statistically significant correlations between the two datasets.

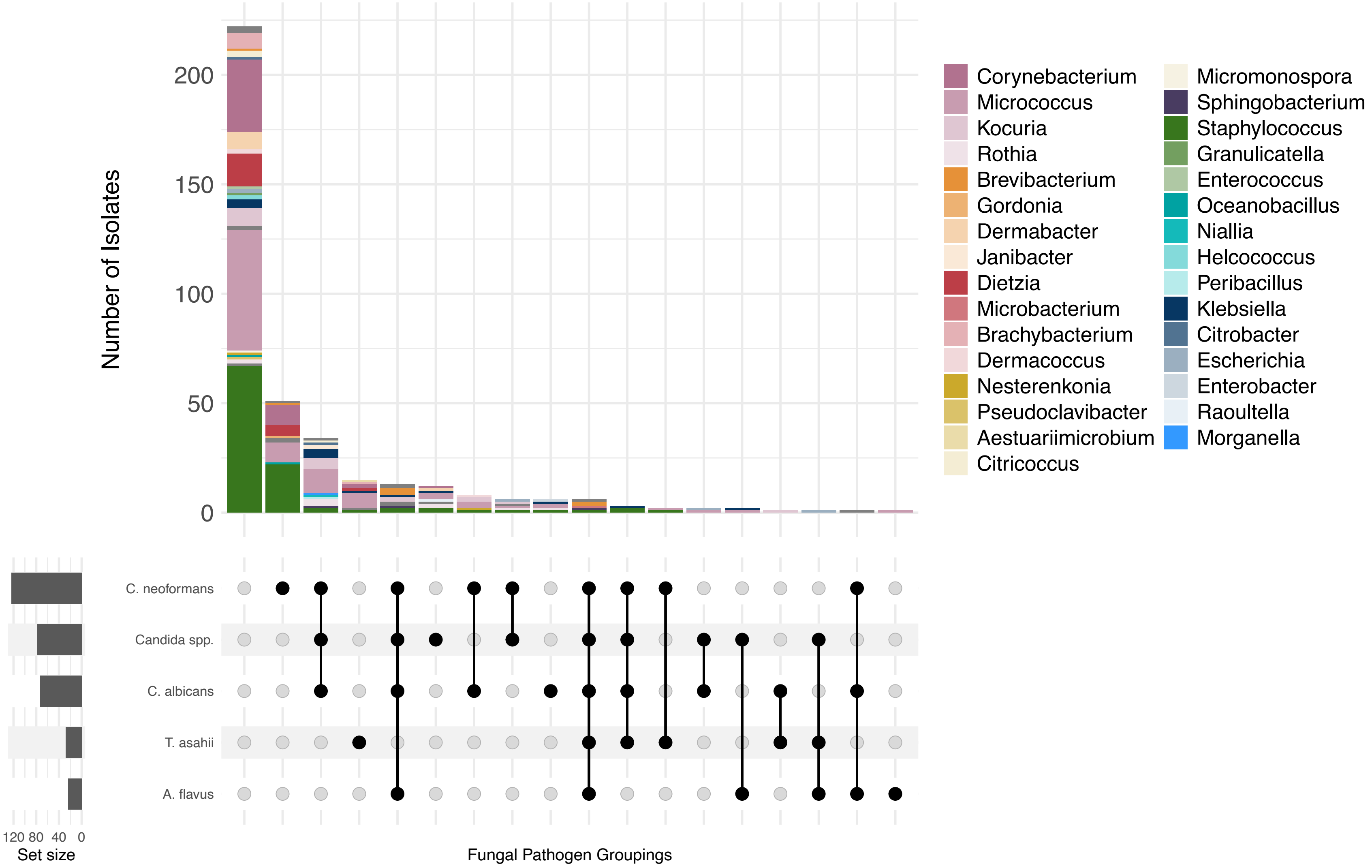

Supplementary Figure S2: Fungal inhibition by skin isolates. The top panel illustrates a stacked bar plot, where each color corresponds to a specific bacterial genus, showing the distribution of strain counts (y-axis) within each genus. The bottom matrix focuses on the intersections among different fungal pathogen types. Rows are labelled to represent distinct fungal pathogens, and each column signifies the overlapping occurrence of these pathogens across sampled sets. Cells within the matrix are filled to illustrate the presence of a fungal pathogen type in the intersecting sets, with filled cells in the same column connected by a horizontal line. To the left of this matrix, bar charts corresponding to the row labels indicate the total number of instances for each fungal pathogen type.

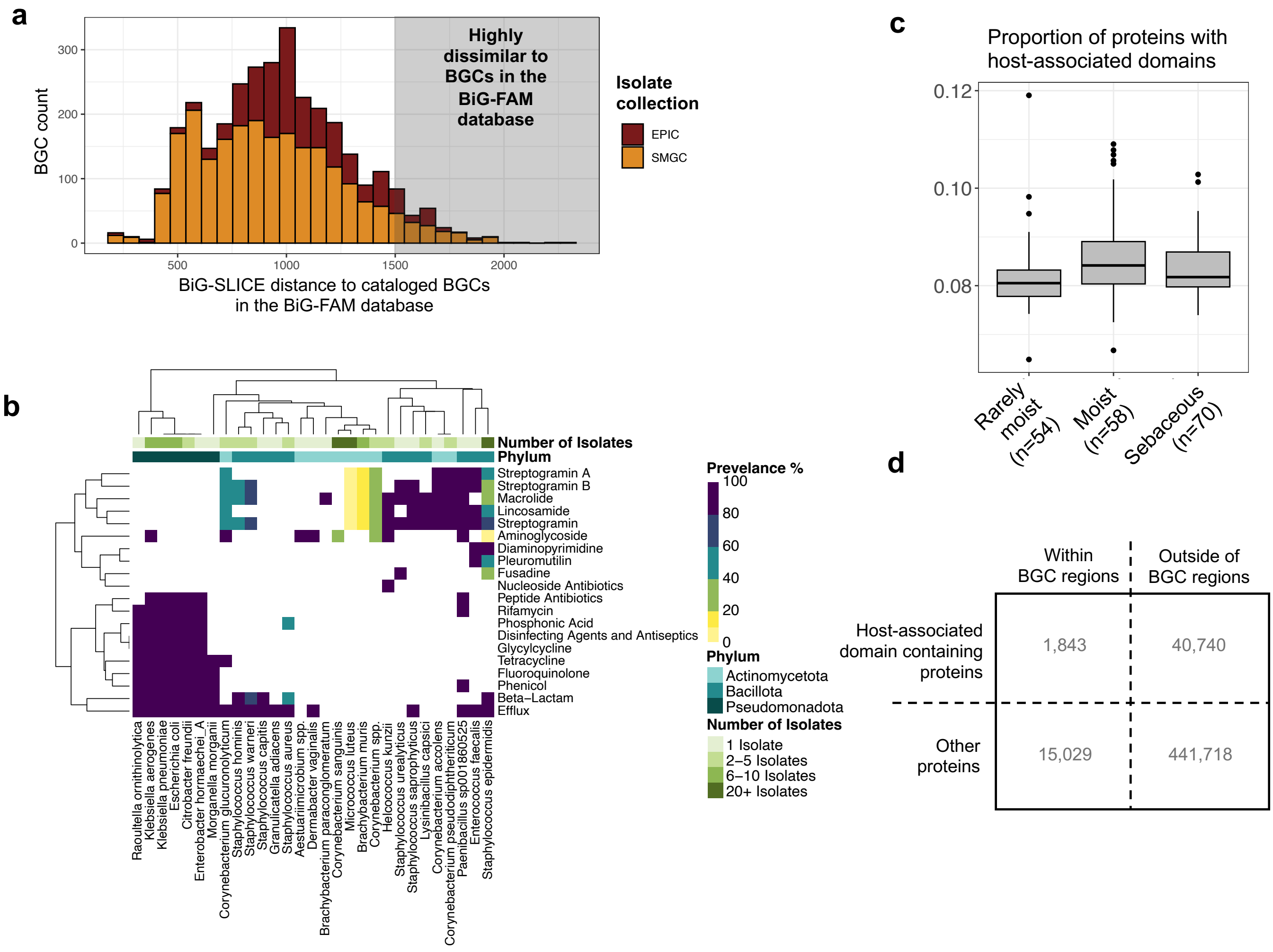

Supplementary Figure S3: Investigations of BGC novelty, antimicrobial resistance, and prevalence of host-associated domains in skin isolate genomes. a, Distance distributions of BGCs from EPIC and SMGC genomes to BGCs cataloged in the BiG-FAM database computed using BiG-SLICE. **b**, Profiling of antibiotic resistance genes in 287 whole genomes from cultured skin isolates. **c**, Comparison of the proportion of proteins with host-associated domains in isolate genomes across different skin site types. Centre lines show the median, box bounds represent the 25th and 75th percentiles, and whiskers demonstrate 1.5×IQR. **d**, A contingency table showing counts of proteins with and without host-associated domains within and outside of predicted BGC regions for EPIC^{HHS} genomes.

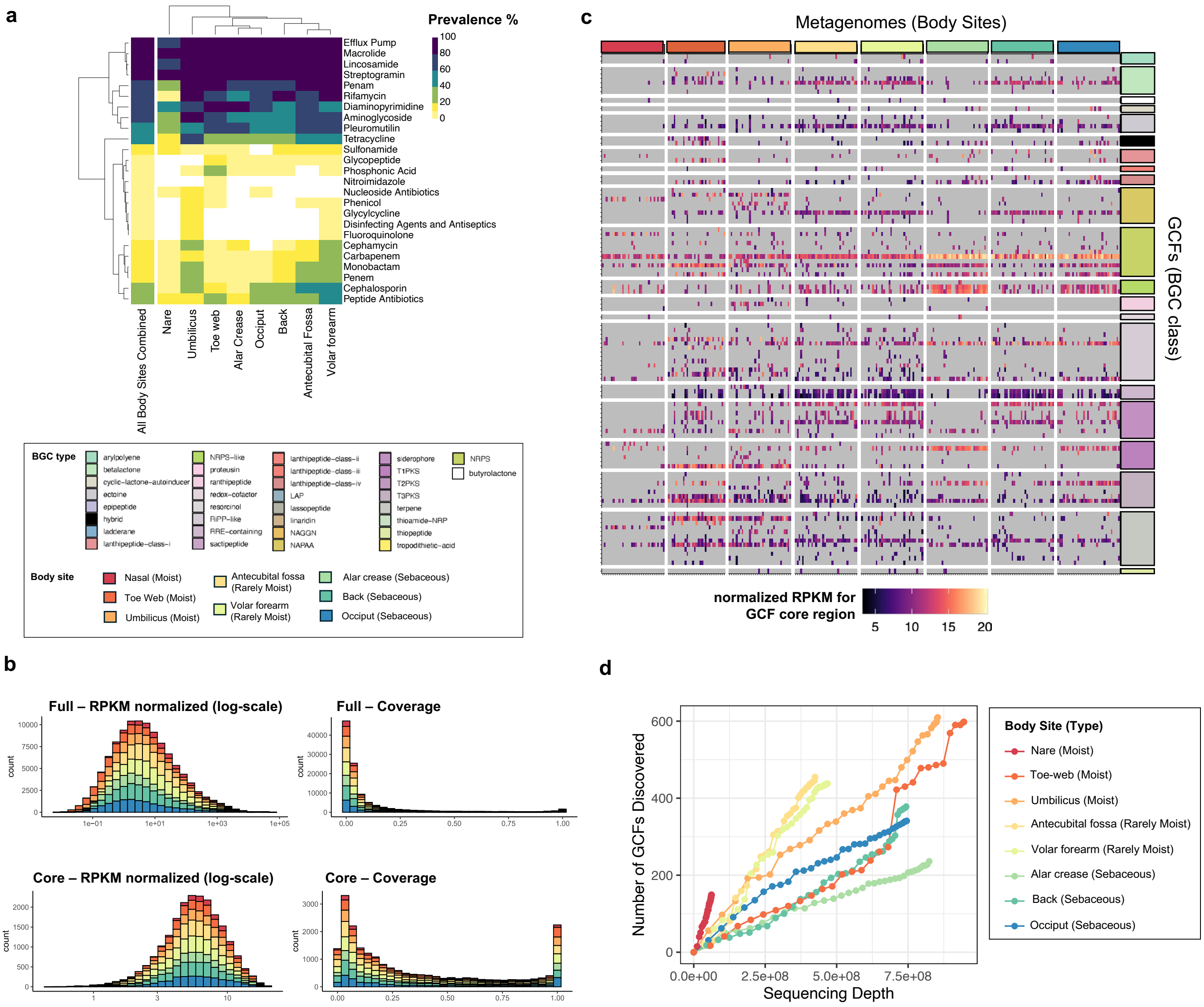

Supplementary Figure S4: Investigations of antimicrobial resistance and BGC distributions across skin metagenomes. a, Prediction of antibiotic resistance genes in the skin metagenomes. Rows indicate antibiotic classes. Columns indicate body sites. Colors indicate prevalence of antibiotic resistance across each body site. **b** Distributions of BiG-MAP's normalized RPKM metric and the proportion of sites covered for GCFs across metagenomes from Swaney et al. 2022 for full and core regions. **c** The normalized RPKM of core regions for GCFs (rows), partitioned by their type (row groups), is shown across metagenomes (columns) divided according to body site of sampling (column groups). Grey indicates the GCF was not detected for a particular metagenome at the required cutoffs. Only GCFs found in five or more metagenomes are shown. RiPP = ribosomally synthesized and post-translationally modified peptide, RRE = RiPP recognition element, LAP = Linear azol(in)e-containing peptides; NRPS = non-ribosomal peptide synthetase, T1PKS = type 1 polyketide synthase, T2PKS = type 2 polyketide synthase, T3PKS = type 3 polyketide synthase, NAPAA = non-alpha poly-amino acid, NAGGN = N-acetylglutaminylglutamine amide. **d** The discovery of distinct GCFs is shown as a function of cumulative sequencing depth across metagenome samples grouped according to body site. Unlike in Fig. 4d where GCF presence was determined within metagenomes using read-alignment and BiG-MAP, here, GCFs were determined from BiG-SCAPE clustering of antiSMASH BGC predictions made on metagenomic assemblies.

Corynebacterium SMGC_7

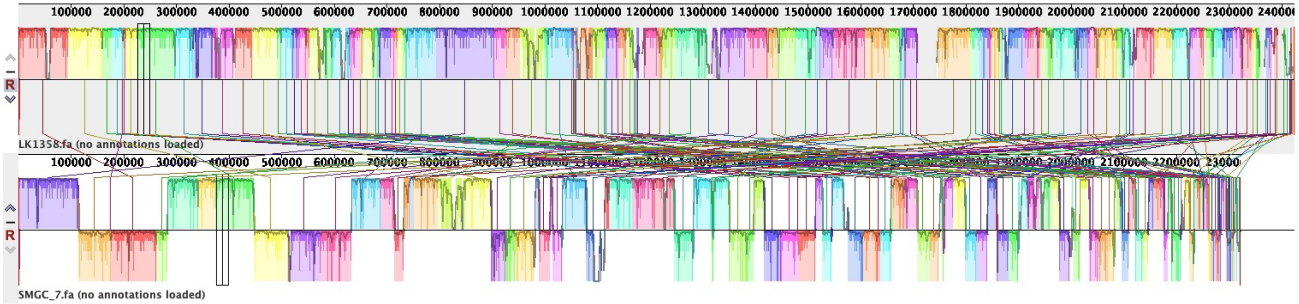

Corynebacterium SMGC_122

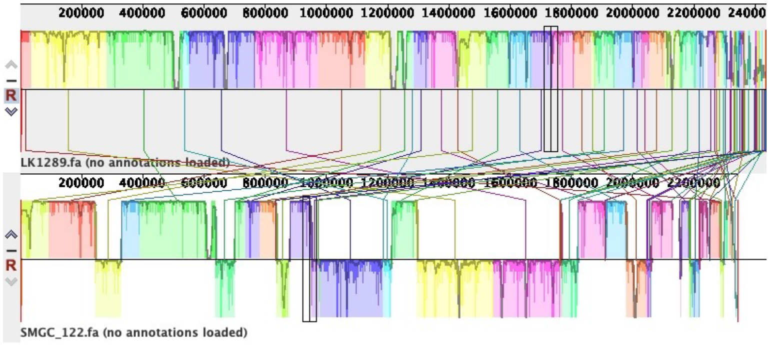

Brachybacterium SMGC_326

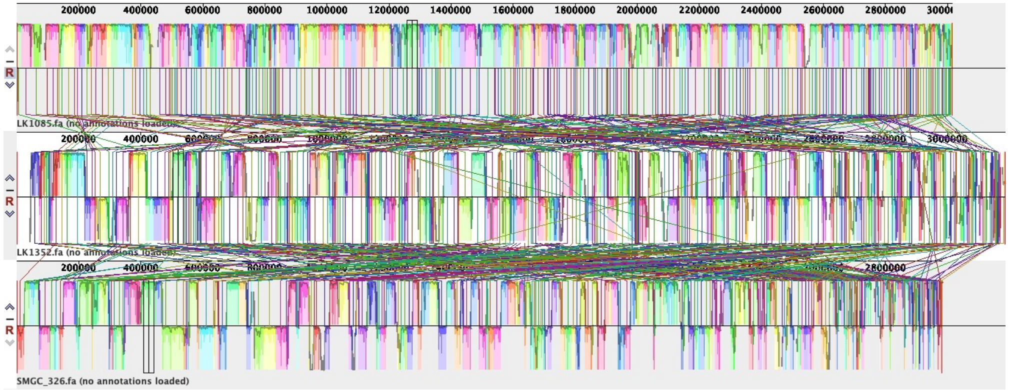

Corynebacterium SMGC_277

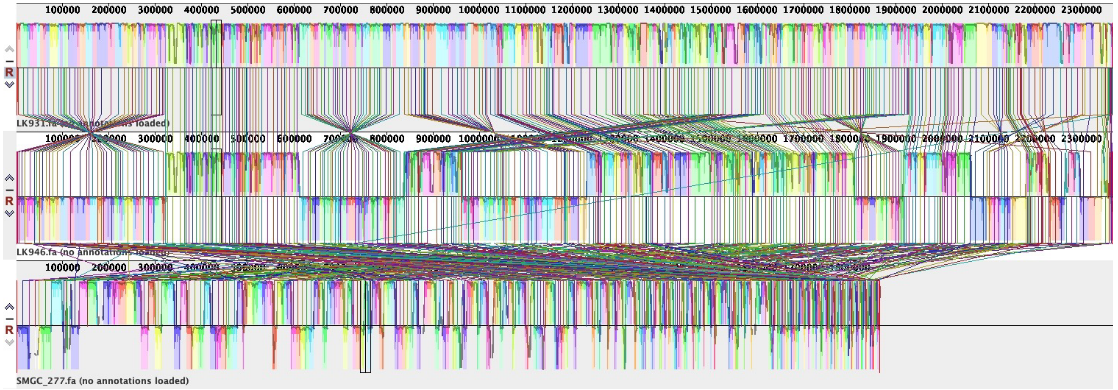

Supplementary Figure S5: Comparative genomics between MAGs and isolate genomes for recently discovered bacterial species from human skin. MAUVE based comparative genomic visualizations of similarities between recently discovered species in the SMGC by Saheb-Kashaf et al. 2022 through metagenomic assembly and isolate genomes determined to belong to the species.

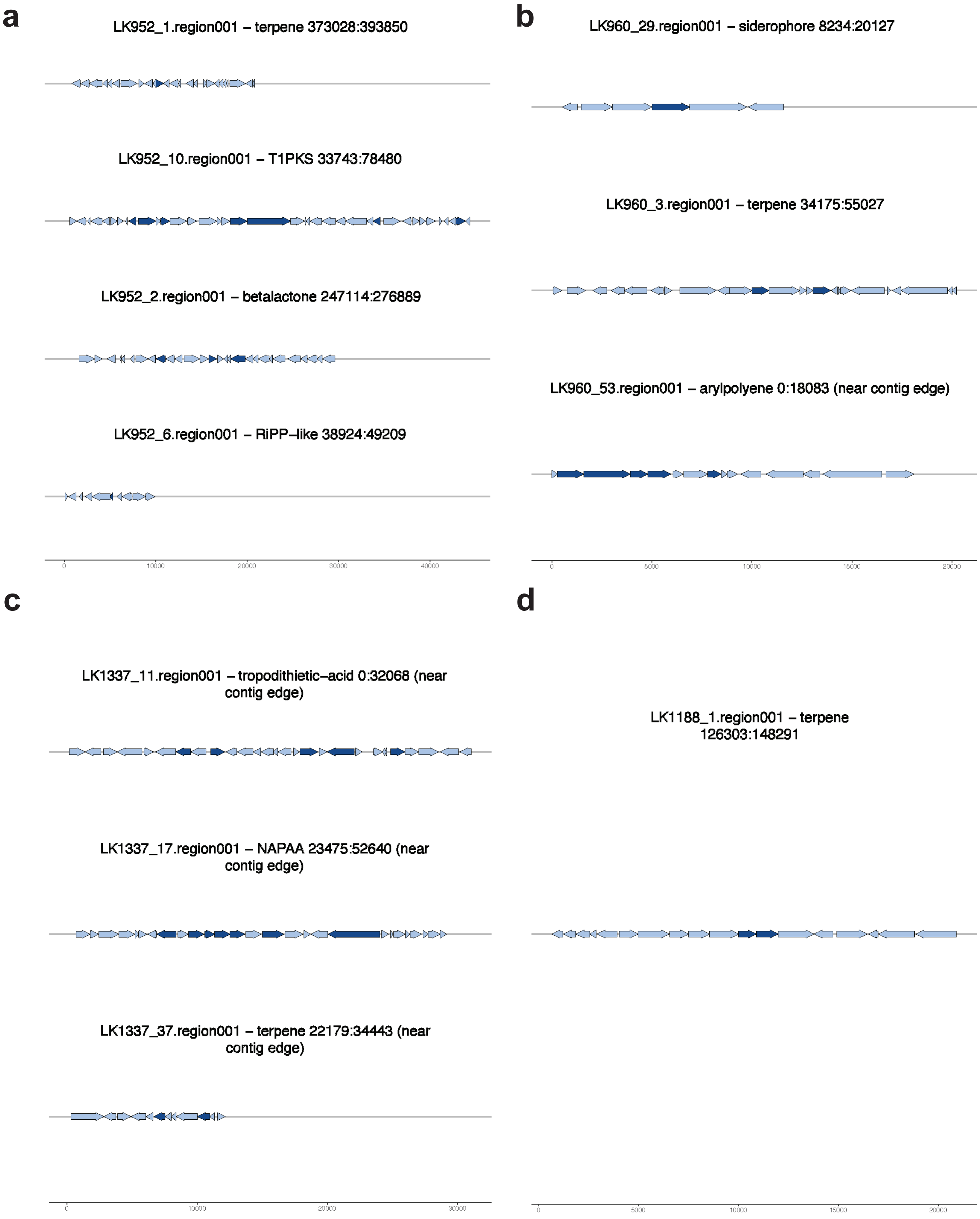

Supplementary Figure S6: Visualization of BGCs in novel, skin-associated species, including **a**, Corynebacterium LK952, **b**, Kocuria LK960, **c**, Brevibacterium LK1337 and **d**, Aestuariimicrobium LK1188. Colors correspond to whether the gene contains a key 'rule'/'detection' domain (dark blue) or not (light blue).
