# Supplementary material for: Large-scale investigation for antimicrobial activity reveals newly-identified defensive species across the healthy skin microbiome
Source: Nat Commun. 2026 May 25;17:6806. doi: 10.1038/s41467-026-73524-z (PMC13385363; doi:10.1038/s41467-026-73524-z)
Supplement: Supplementary file 2 — Description of Additional Supplementary Files [file 41467_2026_73524_MOESM2_ESM.pdf]

Title: Supplementary Dataset 1

Description: Summary statistics for participants providing skin microbiome samples. Demographic characteristics of healthy volunteer participants, stratified by gender. Ethnicity, age range, and median age are reported per group and for the total cohort.

Title: Supplementary Dataset 2

Description: Overview of the isolate collection. Each row represents a unique isolate and features information on its aliases, source, and more. Availability of bioassay data and whole-genome sequence data is also indicated for each isolate. Representative strains were selected following average nucleotide identity (ANI)-based dereplication at a 99% threshold. Taxonomic classification at the genus and species level was assigned using GTDB taxonomy for isolates with genomes or via Sanger sequencing of the 16S region followed by quality trimming and RDP Classifier for genus-level identification for isolates lacking genomes. Initial isolation date and isolation medium refer to the conditions under which the strain was first recovered from the primary sample, while pure culture medium denotes the medium used for routine maintenance. Genome assembly statistics include total genome size (bp), N50 (bp), and number of contigs. Assembly completeness and contamination were assessed using CheckM2, with values reported as percentages.

Title: Supplementary Dataset 3

Description: Overview of novel species discovered. An overview of isolates belonging to novel species or species that were only recently discovered by Saheb Kashaf et al. 2022. For the latter group, information is provided on the relationship of EPIC isolate genomes to MAGs from the SMGC belonging to the same species.

Title: Supplementary Dataset 4

Description: Metagenome-assembled genomes (MAGs) generated from EPIC metagenomes. Columns report the names of metagenomic assembled genomes (MAGs), MAG quality, CheckM2 completeness and contamination, N50, genome size, distinct tRNA and rRNA counts, and information on whether they represent potentially novel species.

Title: Supplementary Dataset 5

Description: Skin isolate inhibition profiles across pathogens. Rows represent pathogen targets, grouped by type and identified by full name and abbreviation. Each column with an isolate identifier corresponds to an isolate, with cell values reporting the bioassay inhibition score.

Title: Supplementary Dataset 6

Description: Inhibition profiles of skin isolates against *C. auris*. Columns report strain ID, host ID, and taxonomic assignment, followed by bioassay inhibition scores from three independent replicates for each isolate.

Title: Supplementary Dataset 7

Description: Overview of BGCs identified by antiSMASH. Each row corresponds to a BGC annotated using antiSMASH along EPIC isolate or SMGC genomes or are MIBiG BGCs that were found to cluster with such BGCs by BiG-SCAPE.

Title: Supplementary Dataset 8

Description: Overview of GCFs from BiG-SCAPE analysis. Each row corresponds to a GCF identified by BiG-SCAPE analysis with information pertaining to BGC type and median length also provided.

Title: Supplementary Dataset 9

Description: GCFs with characterized BGCs from MIBiGv3. Each row corresponds to a GCF which features both a BGC from an EPIC genome and a characterized BGC from MIBiG.

Title: Supplementary Dataset 10

Description: Prediction of antimicrobial resistance traits in the skin isolates. Each row corresponds to potential matches in resistance traits from the CARD to proteins from EPIC isolates with genomes.
