# Supplementary material for: Large-scale investigation for antimicrobial activity reveals newly-identified defensive species across the healthy skin microbiome
Source: Nat Commun. 2026 May 25;17:6806. doi: 10.1038/s41467-026-73524-z (PMC13385363; doi:10.1038/s41467-026-73524-z)
Supplement: Supplementary file 13 — Reporting Summary [file 41467_2026_73524_MOESM13_ESM.pdf]

nature portfolio

Corresponding author(s): Lindsay R. Kalan

Last updated by author(s): Apr 3, 2026

Reporting Summary

Nature Portfolio wishes to improve the reproducibility of the work that we publish. This form provides structure for consistency and transparency in reporting. For further information on Nature Portfolio policies, see our Editorial Policies and the Editorial Policy Checklist.

Statistics

For all statistical analyses, confirm that the following items are present in the figure legend, table legend, main text, or Methods section.

n/a	Confirmed	
☐	☒	The exact sample size (*n*) for each experimental group/condition, given as a discrete number and unit of measurement
☐	☒	A statement on whether measurements were taken from distinct samples or whether the same sample was measured repeatedly
☐	☒	The statistical test(s) used AND whether they are one- or two-sided<br>*Only common tests should be described solely by name; describe more complex techniques in the Methods section.*
☐	☒	A description of all covariates tested
☐	☒	A description of any assumptions or corrections, such as tests of normality and adjustment for multiple comparisons
☐	☒	A full description of the statistical parameters including central tendency (e.g. means) or other basic estimates (e.g. regression coefficient) AND variation (e.g. standard deviation) or associated estimates of uncertainty (e.g. confidence intervals)
☐	☒	For null hypothesis testing, the test statistic (e.g. *F*, *t*, *r*) with confidence intervals, effect sizes, degrees of freedom and *P* value noted<br>*Give P values as exact values whenever suitable.*
☒	☐	For Bayesian analysis, information on the choice of priors and Markov chain Monte Carlo settings
☒	☐	For hierarchical and complex designs, identification of the appropriate level for tests and full reporting of outcomes
☒	☐	Estimates of effect sizes (e.g. Cohen's *d*, Pearson's *r*), indicating how they were calculated

Our web collection on statistics for biologists contains articles on many of the points above.

Software and code

Policy information about availability of computer code

Data collection	Data collection for 16S rRNA sequencing was performed using Sanger sequencing (Functional Biosciences). Metagenomic sequencing was performed on a NovaSeq (Illumina 2 x 150 bp reads).
Data analysis	Genomic data processing, assembly, and dereplication were performed using fastp (v0.20.0), unicycler (v0.4.7), and dRep (v3.2.2), respectively. Taxonomic annotations and validation of novel species relative to newly identified species were performed using GTDB-Tk (v1.7.0) with GTDB release 202 and FastANI (v1.32). Phylogeny construction, filtering, and visualization were performed using GToTree (v1.6.36), PareTree (v1.0.2) and the iTol web application. Biosynthetic gene cluster annotation, clustering across genomes into gene cluster families, conservation assessment across genomes and metagenomes were performed using antiSMASH (v6.0.0), BiG-SCAPE (v1.1.5), BiG-SLICE (v1.1.1), GECCO (v0.9.6), zol (v1.3.11), PyHMMER (v0.11.0), and BiG-MAP (downloaded March 22, 2023 from GitHub). Annotation of antibiotic resistance genes in genomes and metagenomes was performed using RGI (v6.0.1) Assessment of the presence of novel species in public metagenomes was performed using Branchwater (accessed in October 2023). Metagenomic assembly and construction of metagenome-assembled genomes were performed using metaSPAdes (v4.2.0) and MetaWRAP (v1.3.2).

For manuscripts utilizing custom algorithms or software that are central to the research but not yet described in published literature, software must be made available to editors and reviewers. We strongly encourage code deposition in a community repository (e.g. GitHub). See the Nature Portfolio guidelines for submitting code & software for further information.

Data

Policy information about availability of data

All manuscripts must include a data availability statement. This statement should provide the following information, where applicable:

- Accession codes, unique identifiers, or web links for publicly available datasets
- A description of any restrictions on data availability
- For clinical datasets or third party data, please ensure that the statement adheres to our policy

Whole genome assemblies are publicly available from NCBI under BioProject PRJNA803478. Metagenomic sequencing data are publicly available in the Sequence Read Archive (SRA) under BioProject PRJNA763232. Metagenomic assemblies and MAGs can be found on Zenodo (https://zenodo.org/records/18882798). Codes used for analyses and figures are available on GitHub (https://github.com/Kalan-Lab/SkinBioassayStudy) and on Zenodo (https://zenodo.org/records/19012427).

Research involving human participants, their data, or biological material

Policy information about studies with human participants or human data. See also policy information about sex, gender (identity/presentation), and sexual orientation and race, ethnicity and racism.

Reporting on sex and gender	Reporting on sex and gender of the 34 participants who skin microbiome samples were collected from are summarized in Supplementary Table 1.
Reporting on race, ethnicity, or other socially relevant groupings	Race and ethnicity are reported in Supplementary Table 1.
Population characteristics	The population characteristics of the 34 participants who skin microbiome samples were collected and analyzed are detailed in Supplementary Table 1.
Recruitment	Participants were healthy volunteers recruited from UW-Madison. Inclusion criteria included age >18 years old. No formal exclusion criteria were applied. As all participants were mostly recruited from a university laboratory setting, the cohort is likely to be demographically homogeneous, potentially skewing toward a narrow age range, educational background, and occupational environment. Shared occupancy of a laboratory setting may also influence skin microbiome composition through common environmental exposures. Additionally, as participation was voluntary, self-selection bias cannot be excluded. However, as the primary aim of this study was to characterize the diversity and biosynthetic capacity of skin-associated bacteria rather than to draw population-level epidemiological conclusions, these limitations are unlikely to affect the findings.
Ethics oversight	All skin microbiome samples were obtained under an IRB-approved protocol of UW-Madison and informed consent was obtained from all participants. No compensation was given for enrollment in the study.

Note that full information on the approval of the study protocol must also be provided in the manuscript.

Field-specific reporting

Please select the one below that is the best fit for your research. If you are not sure, read the appropriate sections before making your selection.

[x] Life sciences [ ] Behavioural & social sciences [ ] Ecological, evolutionary & environmental sciences

For a reference copy of the document with all sections, see nature.com/documents/nr-reporting-summary-flat.pdf

Life sciences study design

All studies must disclose on these points even when the disclosure is negative.

Sample size	No statistical method was used to predetermine the sample sizes, but our sample sizes were comparable with previous human skin microbiome studies.
Data exclusions	For the bioassay results, we applied a coarse filter for bioassay quality by first removing 70 isolates with inadequate pathogen growth in control wells across the pathogen panel. Subsequently, we filtered for strain redundancy by removing 118 duplicate isolates from the same host sample. Of which, 71 belong to genus Micrococcus and 47 are without identification. 29 isolates with genomes were removed by genome dereplication. Lastly, 12 isolates were removed due to contaminated stocks resulting in discrepancy in identification. After filtering, 386 isolates remained for downstream analysis.
Replication	Large-scale screening data represent one replicate of bioassay results. This approach is consistent with standard practice for large-scale screens, where replication at the screening stage is resource- and time-prohibitive. Reproducibility of findings for the confirmed hits was validated using at least three biological replicates with two technical replicates each. All attempts at replication were successful.
Randomization	Participants were not randomly allocated into experimental groups. Samples were collected from predefined body sites on both left and right sides to reflect different skin microenvironments. For each body site, left-hand side of the participants samples were designated for culture-based analysis and the right side for sequencing. This design was employed to control for inter-individual and inter-site variation, ensuring that culture and sequencing data were derived from anatomically equivalent sites within the sample participant.

Blinding	Blinding was not possible for the in vitro experiments as they were performed by individual investigators who were informed about the experimental groups. Blinding was also not relevant for bioinformatics analyses of the large genomic and bioassay data sets as we performed them using computational tools.

Reporting for specific materials, systems and methods

We require information from authors about some types of materials, experimental systems and methods used in many studies. Here, indicate whether each material, system or method listed is relevant to your study. If you are not sure if a list item applies to your research, read the appropriate section before selecting a response.

Materials & experimental systems

n/a	Involved in the study	
☒	☐	Antibodies
☒	☐	Eukaryotic cell lines
☒	☐	Palaeontology and archaeology
☒	☐	Animals and other organisms
☒	☐	Clinical data
☒	☐	Dual use research of concern
☒	☐	Plants

Methods

n/a	Involved in the study	
☒	☐	ChIP-seq
☒	☐	Flow cytometry
☒	☐	MRI-based neuroimaging

Plants

Seed stocks	The study did not involve any seed stocks.
Novel plant genotypes	No novel plant genotypes were used in the study.
Authentication	This study did not involve any seed stocks or novel plant phenotypes.
