# Supplementary material for: Large-scale investigation for antimicrobial activity reveals newly-identified defensive species across the healthy skin microbiome
Source: Nat Commun. 2026 May 25;17:6806. doi: 10.1038/s41467-026-73524-z (PMC13385363; doi:10.1038/s41467-026-73524-z)
Supplement: Supplementary file 14 — Transparent Peer Review file [file 41467_2026_73524_MOESM14_ESM.pdf]

nature portfolio

Peer Review File

Large-scale investigation for antimicrobial activity reveals newly-identified defensive species across the healthy skin microbiome

Corresponding Author: Dr Lindsay Kalan

This manuscript has been previously reviewed at another journal. This document only contains information relating to versions considered at Nature Communications.

This file contains all reviewer reports in order by version, followed by all author rebuttals in order by version.

Version 0:

Reviewer comments:

Reviewer #2

(Remarks to the Author)
I thank the authors for addressing my comments and I have no further concerns.

(Remarks on code availability)
The code appears to include the necessary information to reproduce their analysis

Open Access This Peer Review File is licensed under a Creative Commons Attribution 4.0 International License, which permits use, sharing, adaptation, distribution and reproduction in any medium or format, as long as you give appropriate credit to the original author(s) and the source, provide a link to the Creative Commons license, and indicate if changes were made.

In cases where reviewers are anonymous, credit should be given to 'Anonymous Referee' and the source.
The images or other third party material in this Peer Review File are included in the article's Creative Commons license, unless indicated otherwise in a credit line to the material. If material is not included in the article's Creative Commons license and your intended use is not permitted by statutory regulation or exceeds the permitted use, you will need to obtain permission directly from the copyright holder.
To view a copy of this license, visit https://creativecommons.org/licenses/by/4.0/
